# FMR1 promotes the progression of colorectal cancer cell by stabilizing EGFR mRNA in an m^6^A-dependent manner

**DOI:** 10.1038/s41419-022-05391-7

**Published:** 2022-11-08

**Authors:** Yuhan Hu, Qingzu Gao, Shuai Ma, Pei Yu, Shuang Ding, Xiaofei Yao, Zheying Zhang, Shuya Lu, Manman Lu, Jinghang Zhang, Yanling Wang, Xinlai Qian, Jiateng Zhong

**Affiliations:** 1 Department of Pathology, School of Basic Medical Sciences, Xinxiang Medical University, Xinxiang, Henan 453000 China; 2 Department of Pathology, The First Affiliated Hospital of Xinxiang Medical University, Xinxiang, Henan 453000 China; 3 Micromorphology Laboratory, School of Basic Medical Sciences, Xinxiang Medical University, Xinxiang, Henan 453000 China; 4 Department of Anesthesiology, The Third Affiliated Hospital of Sun Yat-sen University, Guangzhou, Guangdong China; 5 Department of Pathology, The Third Affiliated Hospital of Xinxiang Medical University, Xinxiang, Henan 453000 P.R. China

**Keywords:** Oncogenes, DNA

## Abstract

FMR1, a new m^6^A reader, is known to be involved in the regulation of cancer progression. However, its role, regulatory mechanism, and clinical significance in colorectal cancer (CRC) are elusive. Here, we showed that FMR1 was upregulated in CRC, and it promoted proliferation and metastasis of CRC cells in vitro and in vivo. Mechanically, FMR1 recognized the m^6^A-modification site in EGFR mRNA, a key molecule in cancer occurrence and targeted therapy, sustained its stability and maintained its expression in an m^6^A-dependent manner, thereby promoting the tumorigenesis and metastasis of CRC. And the effect of FMR1 knockdown in CRC cells could be abolished by METTL3. Furthermore, FMR1 shRNA plasmid carried by attenuated *Salmonella* has an effective anti-tumor effect in vivo. Collectively, we identified the METTL3/FMR1/EGFR axis in the progression of CRC. This novel mechanism indicated that the METTL3/FMR1/EGFR axis is a potential target for early therapeutic intervention in CRC progression.

## Introduction

According to the World Cancer Report 2020, released by the World Health Organization, colorectal cancer (CRC) is the third-most common malignant tumor globally [1]. Although the application of colonoscopies and the improvement of treatment regimens have reduced the mortality of patients with CRC to some extent, the clinical therapeutic effect is still poor in advanced patients [2]. Therefore, in-depth exploration of molecular mechanisms of the occurrence and evolution of CRC and searching for key molecular markers and therapeutic targets have important scientific significance and clinical value for CRC. Epithelial growth factor receptor (EGFR) signaling pathway is frequently activated and participates in CRC progression. EGFR is regarded as a valuable therapeutic target and has attracted much attention in the research and development of tumor-targeted therapeutic drugs [3–5]. Hence, it is of interest to indicate the potential regulatory mechanisms of EGFR pathway in CRC.

The fragile X mental retardation 1 (FMR1) gene is located on human chromosome Xq27.3 and contains seventeen exons, encoding the FMR1 protein (fragile X mental retardation protein 1, FMRP) [6]. The FMR1 protein is an RNA-binding protein (RBP) whose protein structure contains two RNA-binding domains (KH1, KH2) and a C-terminal RG-enriched region involved in RNA binding. It can regulate RNA variable shearing, mRNA stability, and translation and be involved in RNA transport and other important biological processes by binding to the target mRNA coding region or 3′UTR [7]. The loss of the FMR1 protein can cause fragile X syndrome (FXS) and other diseases [8]. Studies revealed that the FMR1 protein plays an important role in the growth and progression of various tumors. Zhu et al. found that the FMR1 protein binds to CCAR1 mRNA and regulates CCAR1 post-transcriptionally, thereby activating the Wnt-signaling pathway and promoting the stemness of HCC cells [9]. Zalfa et al. showed that the FMR1 protein was up-regulated in melanoma and promoted the invasion and metastasis of melanoma by regulating the mRNA of target molecules [10]. Luca et al. discovered that the FMR1 protein can interact with mRNA to regulate the key molecules of epithelial–mesenchymal transition (EMT) post-transcriptionally, ultimately promoting the development of breast cancer [11]. However, whether and how FMR1 regulates CRC tumorigenesis and EGFR signaling pathway remain elusive.

Investigation of the regulatory mechanisms of FMR1 recognized that FMR1 was a new N^6^-methyladenosine (m^6^A) reader [12]. Reports have shown that m^6^A modification could regulate mRNA stability, splicing, and translation [13–15]. m^6^A methylation requires multiple protein complexes in the cell to complete, including writers (METTL3, METTL14, and WTAP), erasers (ALKBH5), and readers, among which METTL3 is involved in cancer progression by regulating the expression of a variety of cancer-related genes [16]. However, the mechanism by which FMR1/METTL3 regulate the EGFR m^6^A methylation and the progression of CRC is still unclear.

Here, we demonstrated that FMR1 is up-regulated in CRC, and the upregulation of FMR1 is significantly correlated to worse clinicopathological characteristics of CRC patients. Meanwhile, we identified that FMR1 could promote the proliferation, cell cycle, and migration of CRC cells. We also found that FMR1 stabilizes EGFR mRNA and facilitates its expression in an m^6^A-dependent manner. The METTL3/FMR1/EGFR signaling axis enriches understanding of CRC progression. Thus, our data suggested that FMR1 is a critical oncogene in CRC development and potential therapeutic target for CRC.

## Materials and methods

### Tissue specimens and cell culture

A total of 120 pairs of CRC tissues and the adjacent normal controls were collected from the First Affiliated Hospital of Xinxiang Medical University (Xinxiang, China) from 2016 to 2017. The tissues were frozen and stored in liquid nitrogen until further use. All the patients were all diagnosed as primary CRC for the first time without radiotherapy, chemotherapy or biological therapy prior to surgery. The present study was performed according to the written approval obtained from the Ethics Committee of Xinxiang Medical University (Xinxiang, China).

The CRC cell lines DLD1, SW480, HT29, HCT116, CACO2, LoVo, HCT8, and RKO were obtained from the American Type Culture Collection (Manassas, VA, USA). All the cells were cultured in RPMI-1640 (HyClone; Logan, UT, USA) supplemented with 10% fetal bovine serum (FBS) (Gibco; Thermo Fisher Scientific, Inc.) at 37 °C with 5% CO_2_.

### Real-time quantitative PCR (RT-qPCR), western blot (WB), and immunohistochemistry (IHC)

The real-time quantitative PCR (RT-qPCR), western blot (WB), and Immunohistochemistry (IHC) were conducted according to previously described methods [17]. Further details are provided in the Supplementary Materials and Methods.

### Lentivirus vectors and plasmids

Lentivirus vectors for FMR1 overexpression or FMR1 knockdown, for METTL3 overexpression, and for EGFR knockdown were all purchased from Genechem Co., LTD. (Shanghai, China).

### Cell counting kit-8 (CCK-8) assay and colony formation assay

The CCK-8 assay and colony formation assay were conducted as previously described [18]. Further details are provided in the Supplementary Materials and Methods.

### Flow cytometry

1 × 10^6^ indicated cells were collected and fixed with 70% cold ethanol. After treated with RNase A (10 μg/mL) for 30 min at 37 °C, the cells were resuspended in 0.5 mL propidium iodide (PI) solution (50 μg/mL in 0.1% sodium citrate with 0.1% NP-40). Cell cycle distribution was analyzed by FACScan cytometry (Becton-Dickinson, San Jose, CA, USA). And a flow cytometer was used to assess cell apoptosis with an Annexin-V-FITC Apoptosis Detection kit (Keygene, Nanjing, China). 24 h after transfection, the cells were harvested and washed twice with cold PBS. Then, 10^6^ cells were resuspended in 200 μl binding buffer supplemented with 10 μl Annexin-V FITC and 5 μl PI. The cells were then incubated in the dark for 10 min. Subsequently, 500 μl binding buffer was added, and a flow cytometric analysis was performed.

### Transwell and wound healing assays

The transwell and wound healing assays were conducted as previously described [19]. Further details are provided in the Supplementary Materials and Methods.

### Nude mice tumorigenicity assay and orthotopic mouse metastatic model

4 to 6-week-old BABL/c female nude mice were purchased from the Center of Laboratory Animal Science of Beijing (Beijing, China). All animal experiments were conducted according to the National Institutes of Health (NIH) Guidelines for Laboratory Animal Care and approved by the Xinxiang Medical University Institutional Animal Care and Use Committee. Xenograft tumors were generated by subcutaneous injection of the indicated cells on the hindlimbs, with 2 × 10^6^ cells for each injection site. The primary tumor growth was measured using a slide caliper and the tumor volume was determined using the formula 1/2 × (length × width^2^). 4 weeks later, all mice were euthanized, the tumors were surgically removed, fixed in neutral buffered 10% formalin, embedded in paraffin, and prepared into 3-μm sections for staining with hematoxylin and eosin (H&E) or immunohistochemical staining.

For orthotopic metastasic assays, the subcutaneous tumors were cut into small masses. Then the healthy 4 to 6-week-old BABL/c female nude mice were anesthetized, and their caeca were exteriorized by laparotomy. The small tumor masses were embedded into the mesentery at the tail end of the cecum. The gut was repositioned to the abdominal cavity and subsequently closed with surgical sutures. Six weeks later, the mice were sacrificed, and all organs were resected for biopsy. All animals were randomized and blinded for experimental grouping.

### Luciferase assays

The Luciferase assays were performed using previously described methods [20]. CRC cells with FMR1 overexpression or the control were seeded in triplicate into 24-well plates (1 × 10^5^ cells per well) and then cultured for 24 h. The constructed GV272 firefly luciferase reporter plasmid of EGFR mRNA 3′ UTR wild type/mutant or the control plasmid was co-transfected into the cells with the reporter Renilla luciferase using Lipofectamine 2000 Reagent (Invitrogen). Luciferase and Renilla activities were detected 24 h after transfection using the Dual-Luciferase Reporter Assay Kit (Promega) according to the manufacturer’s protocol. All experiments were conducted at least three times, and the data are presented as the mean ± SD.

### RNA electrophoretic mobility shift assay (RNA EMSA)

RNA EMSA Kit was purchased from the Axl-bio Technology Company. For the RNA EMSA assay, 5 μl total proteins, 1 μl tRNA, 1 μl RNase Inhibitor and 1 μl FMR1 antibody (Proteintech, USA) were mixed in binding buffer for 10 min at 25 °C. Then the biotin-labeled RNA probes were mixed in binding buffer for 10 min at 25 °C. RNA–protein complexes were separated in 5% of native poly acrylamide gel, transferred to nylon membrane and UV-light crosslinking for 45–60 s. RNA–protein complexes were blotted with HRP-conjugated streptavidin and the final results were visualized by autoradiography.

### RNA immunoprecipitation (RIP)

RNA immunoprecipitation (RIP) experiments were performed with the Magna RIP™ RNA-binding protein immunoprecipitation kit (Millipore) according to protocol [21]. 1 × 10^7^ cells were collected and dissolved in 100% RIP Lysis Buffer with proteinase and RNase inhibitors, and the RIP lysates were incubated with RIP buffer containing magnetic beads conjugated with human anti-FMR1 antibody or nonspecific mouse IgG antibody (Cell Signaling Technology, USA). 24 h later, the RNA/ bead complex was washed five times and resuspended in buffer supplemented with RNase-free DNase and proteinase K. The immunoprecipitated RNAs were subjected to RT-qPCR to detect the enrichment. All tests were repeated three times.

### N^6^-methyladenine RNA-immunoprecipitation (meRIP) followed by RT-qPCR

N^6^-methyladenine RNA-immunoprecipitation was performed using previously described methods [22]. Total RNA was extracted using Trizol reagent (Takara, Japan) and treated with RNase-free DNase I (Roche) to deplete DNA contamination. PolyA RNA was purified and fragmented. 200 μg of fragmented RNA was incubated with 3 μg anti-m^6^A (Synaptic Systems) in RIP buffer (150 mM NaCl, 10 mM Tris and 0.1% NP-40) for 2 h at 4 °C. Then the washed protein A/G magnetic beads (Millipore) were added and the mixture was incubated at 4 °C for further 2 h. Beads were washed 6 times in RIP buffer and incubated with 50 μl immunoprecipitation buffer containing 0.5 mg/ml m6AMP (Sigma-Aldrich) to elute RNA. The immunoprecipitated RNAs were subjected to RT-qPCR to detect the enrichment. All tests were repeated three times.

### Plasmid construction, bacteria, tumor model, and treatment

Three FMR1-specific hairpin RNA (Supplementary Data Table S3) were designed, annealed and ligated into the linearized vector of pGCsilencerU6/Neo, as described previously [23, 24]. The attenuated *S. typhimurium phoP/phoQ-null* strain LH430 was obtained from *S. typhimurium* strain SL1344 by deletion of the *attenuated S. typhimurium phoP/phoQ null strain LH430* locus. The three FMR1 shRNA recombinant plasmids were electroporated into the attenuated *Salmonella*. CT26 cells were injected subcutaneously into BALB/c mice at 1 × 10^6^ cells and started the first treatment after 7 days. Scramble group mice were injected intratumorally with recombinant attenuated *Salmonella* (2 × 10^6^ colony-forming units (CFU) in 100 μl PBS/mouse) and shFMR1 group mice were injected intratumorally with recombinant attenuated *Salmonella* (2 × 10^6^ CFUs in 100 μl PBS/mouse) every 3 days.

### Statistical analysis

Data were analyzed using the Statistical Package for the Social Sciences version 19.0 software (SPSS Inc., Chicago, IL, USA). Quantitative data are presented as the mean ± standard deviation of at least three independent experiments. Student’s *t*-test (two-tailed) or one-way ANOVA were used to calculate the difference between two groups or more than two groups. *χ*^2^ test was used to determine the correlation between FMR1 expression and clinicopathologic features. *p* < 0.05 was considered to indicate a statistically significant difference.

## Results

### The upregulation of FMR1 is correlated with advanced progression of CRC

**Fig. 1:**
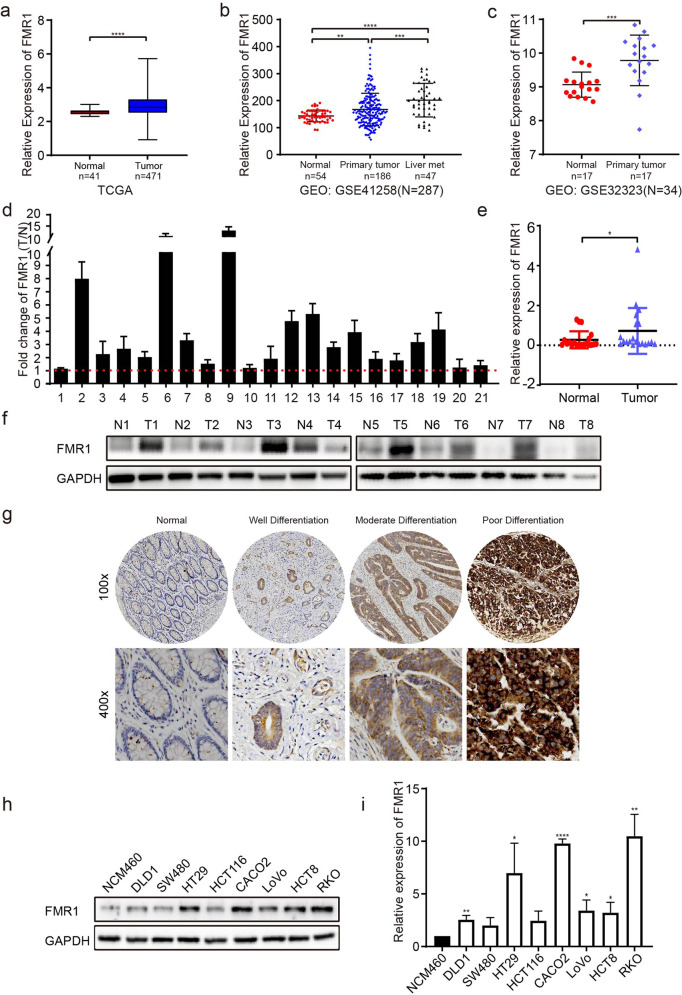
Upregulation of FMR1 is correlated with advanced progression of CRC. **a** The expression of FMR1 in CRC tissues and in normal intestinal mucosal epithelium in the TCGA mRNA sequencing data. **b** The expression of FMR1 in CRC tissues and in normal intestinal mucosal epithelium in the GEO database. **c** The expression of FMR1 in CRC tissues and in normal intestinal mucosal epithelium in the GEO database. **d**, **e** RT-qPCR analysis of FMR1 expression in 21 paired CRC tissues; FMR1 was quantified relative to the matched adjacent no tumor tissues. **f** Western blot analysis of FMR1 expression in 8 paired human CRC tissues. **g** IHC analysis of FMR1 expression in 120 paired paraffin-embedded human CRC tissues (representative results). **h** Western blot analysis of FMR1 expression in CRC cells and the normal intestinal epithelial cell NCM460. **i** RT-qPCR analysis of FMR1 expression in CRC cells and the normal intestinal epithelial cell NCM460. *P < 0.05, **P < 0.01, ***P < 0.001, ****P < 0.0001.

To examine FMR1 expression in CRC tissues and its role in CRC development, we first analyzed the expression of FMR1 by using the data from the Cancer Genome Atlas (TCGA) and GEO (GSE41258 and GSE 32323) public databases. The results showed that the expression of FMR1 was significantly elevated in CRC compared with the normal intestinal mucosal epithelium (Fig. 1a–c). Moreover, the expression of FMR1 was further up-regulated in liver metastases compared with the primary lesions (Fig. 1b). In addition, we detected the expression of FMR1 mRNA and protein in paired fresh CRC tissues from the First Affiliated Hospital of Xinxiang Medical University by RT-qPCR and Western blot. In line with findings from the database, the expression of FMR1 mRNA and protein in CRC tissues were a strong overexpression in a subset of patients compared with paired normal intestinal mucosa adjacent to cancer (Fig. 1d–f). Immunohistochemistry (IHC) was further used to detect the expression level of FMR1 protein in 120 paired CRC paraffin samples, and the results revealed that the expression of FMR1 protein in CRC tissues was highly enhanced compared with normal adjacent intestinal mucosa tissues (Fig. 1g). Moreover, its expression was positively correlated with tumor size, degree of differentiation, TNM stage, and metastasis in CRC patients (Table 1, Fig. 1g). Meanwhile, RT-qPCR and Western blot analyses were performed to assess the expression of FMR1 in 8 CRC cell lines, including DLD1, SW480, HT29, HCT116, CACO2, LoVo, HCT8, RKO and the normal intestinal mucosal epithelial cell NCM460. The results revealed that FMR1 was up-regulated in CRC cells (Fig. 1h, i). Together, these results suggest that FMR1 is overexpressed in CRC and correlated with advanced progression in CRC patients.

**Table 1:** The relationship between FMR1 expression and clinicopathological parameters.

Clinicopathological variables	FMR1 expression		*χ*^2^	*P* value
	High 60	Low 60		
*Age*^a^				
≤mean(58)	27	28	0.034	0.855
>mean(58)	33	32		
*Gender*				
Male	28	30	0.133	0.715
Female	32	30		
*Diameter (cm)*^b^				
≤4.5	24	35	4.034	0.045*
>4.5	36	25		
*TNM classification*				
I–II	34	47	6.420	0.011*
III–IV	26	13		
*Differentiation*				
Well	9	20	7.212	0.027*
Moderate	36	33		
Poor	15	7		
*Metastasis*				
No	46	55	5.065	0.024*
Yes	14	5		

^a^Group of age was performed according to median.

^b^Diameter was grouped according to median.

*p < 0.05.

Bold values indicates statistical difference between the groups.

### FMR1 regulates proliferation, apoptosis and cell cycle of CRC cells

**Fig. 2:**
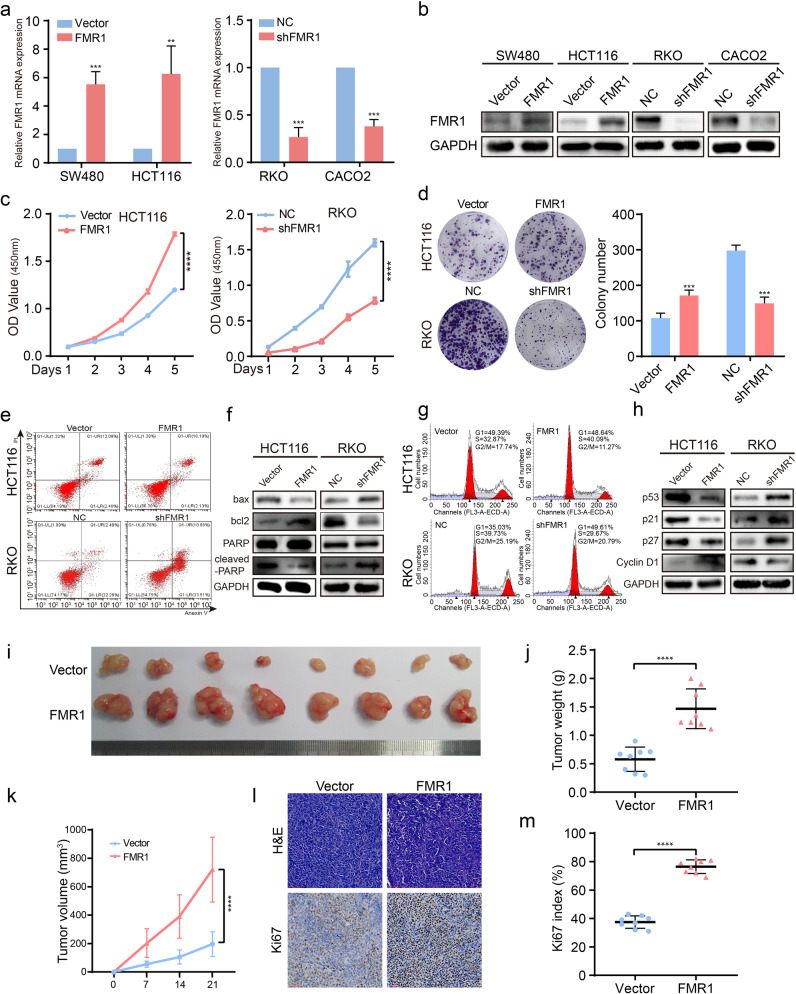
FMR1 regulates proliferation, apoptosis, and cell cycle of CRC cells in vitro and in vivo. **a** RT-qPCR was used to validate the expression of FMR1 in SW480 and HCT116 CRC cells with FMR1 stable overexpression and knockdown. **b** Western blot was used to validate the expression of FMR1 in CRC cells with FMR1 stable overexpression and knockdown. **c** CRC cell proliferation was analyzed by CCK8 assays. **d** Representative results of colony formation; the numbers of colonies containing >50 cells were scored. The number of colonies counted was of an entire well and the error bars represent mean ± SD from three independent experiments. **e** Apoptosis assay by flow cytometry. Annexin-positive/PI-negative (right lower quadrant) cells were analyzed for apoptosis rate. **f** Western blot was used to test the molecular markers of apoptosis. **g** Flow-cytometry analyses of the cell cycle of the indicated CRC cells. **h** Western blot was used to test the molecular markers of cell cycle. **i** The xenograft models were generated after injecting HCT116/Vector and HCT116/FMR1 cells in nude mice (n = 8/group). **j** The tumor weight was measured after the nude mice were euthanized. Error bars represent the means ± SD. **k** The tumor volumes were measured on the indicated days. The data points represent the mean tumor volumes ± SD. **l** The sections of tumor were subjected to H&E staining or IHC staining using an antibody against Ki-67. **m** Ki67 index was calculated. The data points represent the mean tumor volumes ± SD. **P < 0.01, ***P < 0.001, ****P < 0.0001.

To explore whether FMR1 regulated the CRC cells phenotype, we first overexpressed FMR1 in SW480 and HCT116 cells and suppressed its expression in RKO and CACO2 using a lentiviral delivery. The results of Western blot and RT-qPCR analyses indicated that stable cell lines were successfully constructed (Fig. 2a, b). We used CCK-8 and colony formation assays to access the effects of FMR1 on CRC cell proliferation in vitro. The results revealed that overexpression of FMR1 promoted the proliferative ability of CRC cells, while the cell proliferation ability was repressed when FMR1 was inhibited (Fig. 2c, d and Supplementary Fig. S1a, b).

Subsequently, the effect of FMR1 on apoptosis of CRC cells was analyzed by flow cytometry and Western blot. The results showed that the rate of apoptosis was decreased when FMR1 was overexpressed, but the rate of apoptosis was higher when FMR1 was inhibited (Fig. 2e and Supplementary Fig. S1c). Consistent with flow cytometry, the results of Western blot revealed that the anti-apoptotic protein bcl2 was increased and the pro-apoptotic protein bax and cleaved-PARP were decreased when FMR1 was overexpressed, and vice versa (Fig. 2f and Supplementary Fig. S1d). Furthermore, the distribution of CRC cells within the stages of the cell cycle was examined by flow cytometry. Cells with FMR1 overexpression showed a decrease in the percentage of cells in the G1/G0 peak and an increase in the percentage of cells in the S peak; however, CRC cells treated with FMR1 inhibition showed an increase in the percentage of cells in the G1/G0 peak and a decrease in the percentage of cells in the S peak (Fig. 2g and Supplementary Fig. S1e). The results of Western blot also indicated that cyclin D1 was up-regulated, while p53, p21, and p27 were down-regulated when FMR1 was overexpressed, and vice versa (Fig. 2h and Supplementary Fig. S1f).

To confirm the previous results in an in vitro experiment, CRC cells with FMR1 stably overexpressed were injected into the dorsal subcutaneous tissues of nude mice, and the volumes of the tumors formed by CRC cells with FMR1 overexpression grew faster than those formed by control cells (Fig. 2i, j). Moreover, the tumor weight and percentage of Ki67-positive cells in subcutaneous tumors of FMR1 overexpression was higher (Fig. 2k–m). Collectively, these results demonstrate that FMR1 could promote CRC cell proliferation by prohibiting apoptosis and promoting cell cycle progression.

### FMR1 facilitates the metastasis of CRC cells in vitro and in vivo

**Fig. 3:**
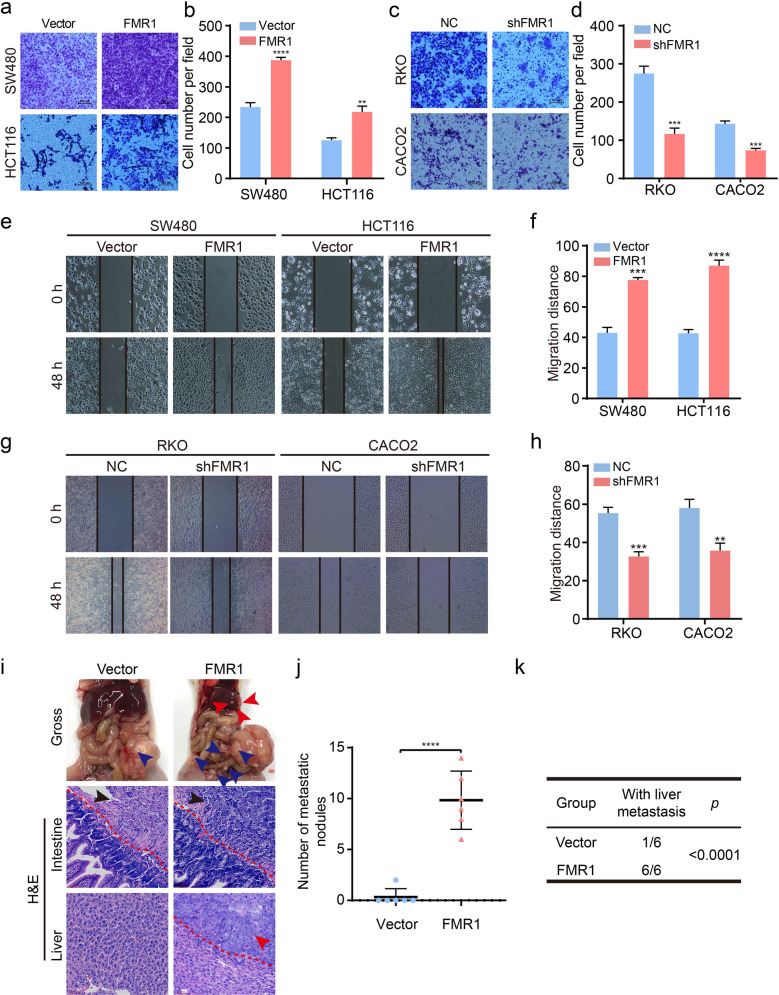
FMR1 facilitates the metastasis of CRC cells in vitro and in vivo. **a**–**d** Representative results of transwell assays. The bar chart represents the migration cell numbers. Error bars represent the means ± SD of 3 different fields. **e**–**h** Representative results of wound healing assays. The bar chart represents the migration distance. Error bars represent the means ± SD of 3 different fields. **i** Representative gross images of the orthotopic mouse metastatic model from different experimental groups are shown. Sections of the liver were stained with H&E. **j** The statistical analysis of number of liver metastatic nodules. Error bars represent the means ± SD. **k** The statistical distribution of metastasis numbers. **P < 0.01, ***P < 0.001, ****P < 0.0001.

We then performed transwell assays to examine the influence of FMR1 on the migration of CRC cells in vitro. The results showed that FMR1 overexpression remarkably enhanced the migration capacity of CRC cells, however, this phenomenon was remarkably reversed when FMR1 was inhibited (Fig. 3a–d). Consistent with this result, the results of wound healing assays also revealed that FMR1 overexpression could promote the migration of CRC cells, and vice versa (Fig. 3e–h). Next, we used an orthotopic mouse metastatic model to examine the metastasis potential with FMR1-overexpressing HCT116 cells. The numbers of tumor nodules in liver metastasis increased significantly when FMR1 was overexpressed as compared with the control group (Fig. 3i–k). Thus, these results reveal that FMR1 enhances metastasis of CRC cells.

### FMR1 targets EGFR in the progression of CRC

**Fig. 4:**
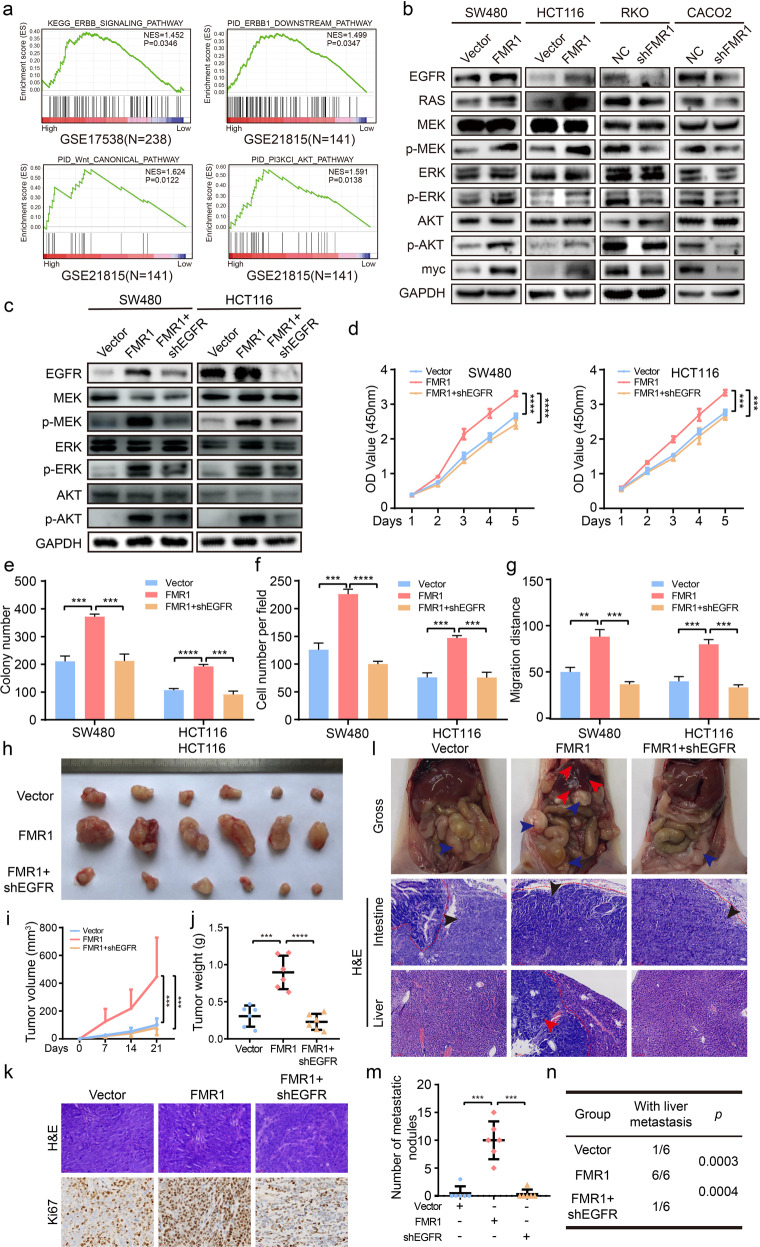
FMR1 promotes the progression of CRC by upregulating EGFR. **a** GSEA plot showed that high expression of FMR1 was positively correlated with the ERBB and its downstream signaling pathway in published CRC patient gene expression profiles (NCBI/GEO/GSE17538 and GSE21815). **b** Western blot was used to test the molecular markers of ERBB and its downstream pathway. **c** Western blot was used to validate the CRC cells with EGFR stably knockdown. **d** CRC cell proliferation was analyzed by CCK8 assays. **e** The results of colony formation; the numbers of colonies containing >50 cells were scored. The number of colonies counted was of an entire well and the error bars represent mean ± SD from three independent experiments. **f** The results of transwell assays. The bar chart represents the migration cell numbers. Error bars represent the means ± SD of 3 different fields. **g** The results of wound healing assays. The bar chart represents the migration distance. Error bars represent the means ± SD of 3 different fields. **h** The xenograft models were generated after injecting Vector, FMR1 and FMR1/shEGFR cells in nude mice (n = 6/group). **i** The tumor volumes were measured on the indicated days. The data points represent the mean tumor volumes ± SD. **j** The tumor weight was measured after the nude mice were euthanized. Error bars represent the means ± SD. **k** The sections of tumor were subjected to H&E staining or IHC staining using an antibody against Ki-67. **l** Representative gross images of the orthotopic mouse metastatic model from different experimental groups are shown. Sections of the liver were stained with H&E. **m** The statistical analysis of number of liver metastatic nodules. Error bars represent the means ± SD. **n** The statistical distribution of metastasis numbers. **P < 0.01, ***P < 0.001, ****P < 0.0001.

To explore the underlying mechanisms of FMR1 in CRC development, we first analyzed FMR1-regulated gene signatures via gene set enrichment analysis (GSEA). The results revealed that a higher expression of FMR1 was positively correlated with an enrichment of ERBB signaling pathway and its downstream pathways including the ERBB1 downstream pathway, Wnt_CANONICAL pathway, and PI3KCI_AKT pathway (GSE17538, GSE21815; Fig. 4a). The results of Western blot also showed that the molecular markers EGFR, RAS, p-MEK, p-ERK, p-AKT, and myc were up-regulated when FMR1 was overexpressed, however, the expression of these signatures were down-regulated when FMR1 was inhibited (Fig. 4b).

To examine the contribution of EGFR to the promoting effects of FMR1 on CRC cell proliferation and migration, we performed rescue experiments. We down-regulated the level of EGFR expression in FMR1-overexpressing CRC cells by transfecting the lentivirus vector. The expression levels of EGFR, p-MEK, p-ERK, and p-AKT were confirmed by Western blot (Fig. 4c). It showed that the upregulation of these signatures caused by FMR1 overexpression could be reversed with EGFR knockdown. The results of functional experiments in vitro also showed that EGFR inhibition could significantly reverse the promoting effects of FMR1 on CRC cell proliferation and migration in vitro by CCK8, colony formation, transwell, and wound healing assays (Fig. 4d–g; Supplementary Fig. S2a–c).

Then, we performed an in vivo tumourigenesis experiment in nude mice. The results showed that tumor volumes and weights were significantly increased when FMR1 was overexpressed, though this phenomenon could be reversed by EGFR knockdown (Fig. 4h–j). EGFR could also reverse the Ki-67 index in xenografts raised by FMR1 overexpression (Fig. 4k). Moreover, we established a liver metastasis model in nude mice and found that EGFR inhibition could decrease the number of definite liver colonization sites that could be increased by FMR1 overexpression (Fig. 4l–n). These data indicated that EGFR is specifically required for FMR1 to regulate cellular phenotypes in CRC cells.

### FMR1 sustains EGFR mRNA stability in an m^6^A-dependent manner

**Fig. 5:**
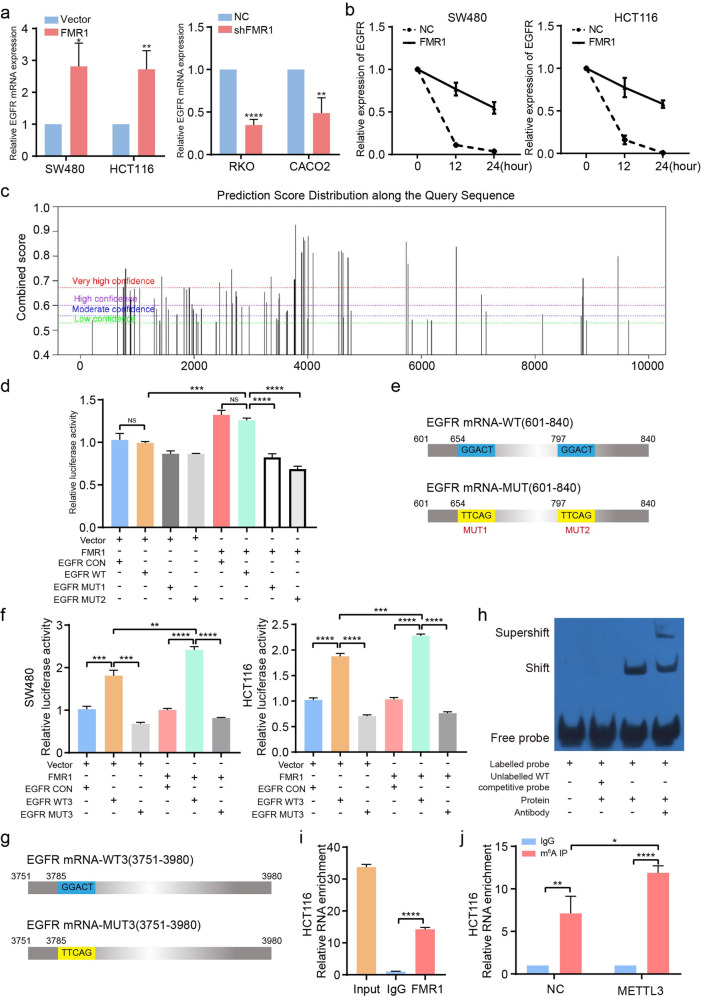
FMR1 regulates EGFR mRNA stability via m^6^A-dependent manner. **a** RT-qPCR was used to test the expression of EGFR when FMR1 was stably overexpressed or knockdown. **b** RT-qPCR was used to test the stability of EGFR mRNA in the indicated CRC cells. **c** Prediction of specific binding sites recognized by FMR1 protein on the EGFR mRNA sequence. **d** Luciferase activity assay showed that FMR1 protein influences the luciferase activity of EGFR mRNA (654 and 797 position) in SW480 and HCT116 cells. **e** Schematic representation of wild-type (EGFR mRNA-wt) and mutant (EGFR mRNA-mut) EGFR mRNA constructs. **f** Luciferase activity assay showed that FMR1 protein influences the luciferase activity of EGFR mRNA (3785 position which is locating on the EGFR 3'UTR) in SW480 and HCT116 cells. **g** Schematic representation of wild-type (EGFR mRNA-wt) and mutant (EGFR mRNA-mut) EGFR mRNA constructs. **h** The binding between FMR1 protein and EGFR mRNA (3785 position) was validated by RNA EMSA. **i** RIP-derived RNA in HCT116 cells were measured by RT-qPCR. **j** Gene-specific m^6^A qPCR validation of m^6^A levels of EGFR mRNA (3785 position) in HCT116 cells. *P < 0.05,**P < 0.01, ***P < 0.001, ****P < 0.0001. NS not significant.

It has been demonstrated FMR1 can regulate protein expression through targeted binding and stabilization of mRNA [25]. We initially examined the effect of FMR1 on the EGFR mRNA expression. RT-qPCR showed that the expression of EGFR mRNA increased when FMR1 was up-regulated in CRC cells, and vice versa (Fig. 5a). Meanwhile, we treated CRC cells with the transcriptional suppressor Actinomycin D. At 0, 12, and 24 after the treatment, RNA was extracted and RT‐qPCR was performed. We found that CRC cells with FMR1 overexpression showed an increase in EGFR mRNA levels compared with the control group at the indicated time points, proving that FMR1 could regulate its stability (Fig. 5b).

It is recognized that FMR1 was a new m^6^A reader that could recognize the m^6^A site containing the sequences “GGAC” or “ACU” [12]. We predicted the m^6^A modification sites that FMR1 could recognize in the full-length mRNA sequence of EGFR using the SRAMP website (http://www.cuilab.cn/sramp/). The results showed that there were multiple m^6^A modification sites recognized by FMR1 protein in an EGFR mRNA sequence with very high confidence (Fig. 5c and Supplementary Fig. S3a). We chose three positions (654, 797, and 3758) with the sequence “GGACU,” which was the most likely FMR1-binding sites and had a much higher score for further investigation. Subsequently, we designed EGFR-WT/MUT1/MUT2 (601–840) which encompassed the binding sites 654/797 and EGFR-WT3/MUT3 (3751–3780) which encompassed the binding site 3758, and conducted a dual‐luciferase reporter assay (Fig. 5d–g). The dual-luciferase assay showed that FMR1 overexpression significantly increased the luciferase activity of EGFR mRNA containing the 3758 binding site which is locating on the EGFR 3′UTR, whereas the promoting effect was attenuated when the site was mutant (Fig. 5f, g). Although the luciferase activity of EGFR mRNA containing 654/797 changed when the two binding sites were mutant, it did not apparently change when FMR1 was overexpressed (Fig. 5d, e). These results suggested that FMR1 may recognize EGFR mRNA mainly by binding to the 3758 binding site. RNA EMSA and RIP also demonstrated that FMR1 could bind to the 3758 binding site in EGFR mRNA (Fig. 5h, i). We then investigated the m^6^A modification status of the 3758 binding site in EGFR mRNA by performing meRIP-qPCR assay. We confirmed that there was an obvious m^6^A modification of this position, and overexpression of m^6^A writer METTL3 increased its m^6^A modification level (Fig. 5j). Taken together, the results demonstrated that FMR1 promotes stabilization and expression of EGFR mRNA by an m^6^A-dependent manner.

### FMR1, METTL3, and EGFR jointly regulate the progression of CRC cells

**Fig. 6:**
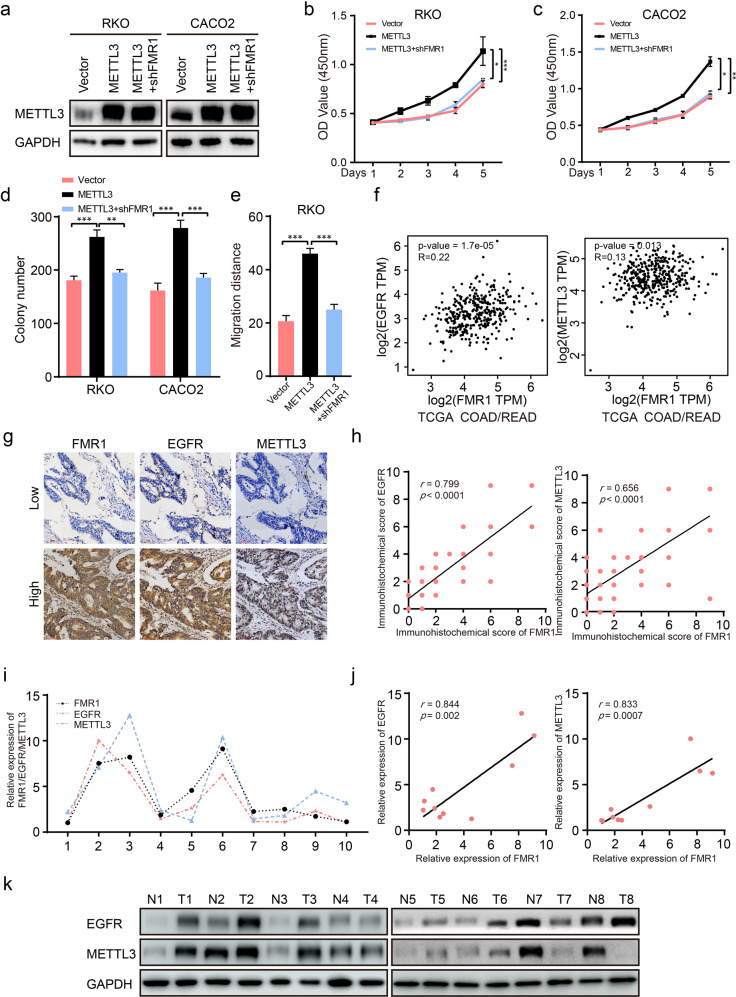
FMR1, METTL3, and EGFR work together to regulate the progression of CRC cells. **a** Western blot was used to validate the expression of METTL3 in RKO, RKO/shFMR1, CACO2, and CACO2/shFMR1 CRC cells with METTL3 stable overexpression. **b**, **c** CRC cell proliferation was analyzed by CCK-8 assays. **d** The results of colony formation; the numbers of colonies containing >50 cells were scored. **e** The results of wound healing assays. The bar chart represents the migration distance. Error bars represent the means ± SD of 3 different fields. **f** Pearson’s correlation analysis of FMR1 and EGFR/METTL3 in CRC tissues. The data was provided by the website GEPIA, which provides pair-wise gene expression correlation analysis for given sets of TCGA expression data. **g** The FMR1 expression was positively associated with EGFR and METTL3 in 120 CRC arrays. Two representative cases are shown. **h** Correlation analysis of the immunohistochemistry score of FMR1 and EGFR/METTL3 in 120 specimens. **i**, **j** RT-qPCR analysis of FMR1, EGFR and METTL3 expression in 10 paired human colorectal cancer tissues and adjacent normal tissues, the results are presented as the fold change in tumor tissues relative to the matched adjacent normal tissues. Spearman correlation analysis showed a positive relationship between FMR1 expression level and EGFR/METTL3 mRNA in 10 CRC tissues. **k** Western blot analysis of EGFR and METTL3 expression in 8 paired human CRC tissues. *P < 0.05, **P < 0.01, ***P < 0.001.

METTL3 is a known m^6^A writer that can facilitate tumor progression via an m^6^A-dependent mechanism in CRC [26]. To validate whether FMR1 promotes CRC cell proliferation and metastasis by recognizing m^6^A modification site, we constructed stable CRC cell lines with METTL3 overexpression and stable CRC cell lines with METTL3 overexpression and FMR1 inhibition. We used Western blot to confirm the stable CRC cell lines (Fig. 6a). The results of CCK-8 and colony formation assays showed that METTL3 could promote CRC cell proliferation; however, the effect could be reversed when FMR1 was knockdown (Fig. 6b–d and Supplementary Fig. S4a). The results of wound healing assay also showed that FMR1 inhibition could reverse the promoting effect of METTL3 on cell migration (Fig. 6e and Supplementary Fig. S4b).

Then we used the online web service GEPIA to analyze the expression correlation between FMR1 and EGFR/METTL3 in CRC tissues from TCGA database. It was demonstrated that there was a positive correlation between FMR1 and EGFR/METTL3 (Fig. 6f). Subsequently, an analysis of 120 CRC tissue chip specimens by IHC revealed that the expression of FMR1 was positively correlated with that of EGFR and METTL3 (Fig. 6g, h, *r* = 0.799, *P* < 0.001; *r* = 0.656, *P* < 0.001). We also tested the EGFR protein level in FMR1-overexpressing xenografts by IHC. This revealed that the expression of EGFR protein increased when FMR1 was overexpressed (Supplementary Fig. S4c). Consistently, FMR1 was positively correlated with the levels of EGFR/METTL3 mRNA in 10 cases of clinical CRC tissues (Fig. 6i, j, *r* = 0.844, *P* = 0.002; *r* = 0.833, *P* < 0.001). Moreover, we detected the expression of EGFR and METTL3 protein in paired fresh CRC tissues which were used to test the expression of FMR1 by Western blot. The results showed that the expression of EGFR and METTL3 were all increased compared with paired normal intestinal mucosa adjacent to cancer (Fig. 6k).

### Anti-tumor effect of FMR1 shRNA carried by attenuated *Salmonella* in vivo

**Fig. 7:**
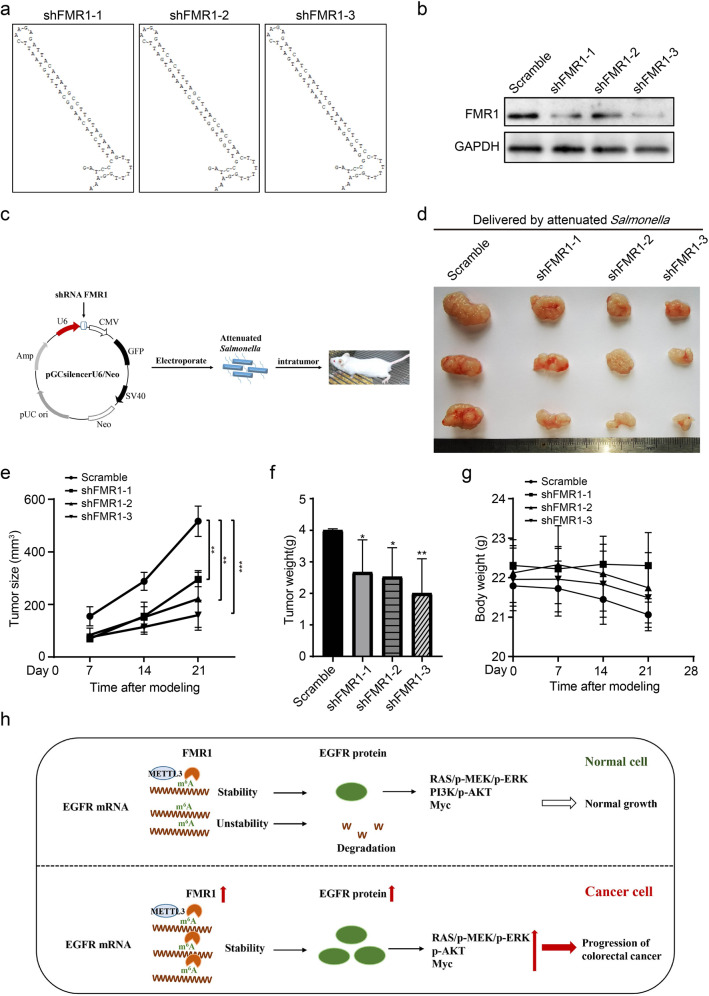
FMR1 shRNA carried by attenuated *Salmonella* can effectively cure colon cancer in vivo. **a** Local secondary structure of FMR1 mRNA at the regions targeted by the three FMR1 small interfering RNAs. **b** Western blot was used to validate the expression of FMR1 in CT26 cells with FMR1 shRNA. **c** Schematic of subcutaneous tumor formation assay in CT26 mice formed by CRC cells with FMR1 shRNA carried by attenuated *Salmonella*. **d** Images of the representative tumors in each group on posttreatment 14 days. **e** Tumor size analysis. **f** Tumor weight analysis. **g** Body weight analysis. **h** Proposed model underlying the effect of FMR1-medicated EGFR mRNA stability in colorectal cancer. Data are shown as means ± SD. *P < 0.05, **P < 0.01, ***P < 0.001.

On the basis of the above results, we used our RNA interference drug platform to further explore the therapeutic application of FMR1. We designed three FMR1 shRNA sequences and inserted them into the pSilencer plasmid, as described above [24] (Fig. 7a). Western blot results revealed that the three shRNAs have a good effect on inhibiting the expression of FMR1 (Fig. 7b). Using mouse colon cancer CT26 cells to establish a subcutaneous animal model, the results of intratumoral injection treatment showed that FMR1 shRNA carried by attenuated *Salmonella* can effectively inhibit tumor growth and has no significant effect on the animal’s body weight (Fig. 7c–g). The results suggested that the RNA interference scheme targeting FMR1 has great potential in the treatment of colon cancer.

## Discussion

FMR1 is a RBP that regulates gene transcription at the post-transcriptional level by interacting with RNA, thus playing an important role in the occurrence and development of tumors [27, 28]. Recently, Di Grazia et al. have reported that the FMR1 protein could regulate RIPK1 and CRC resistance to necroptosis [29]. However, its roles and the underlying mechanisms in the progression of CRC are largely unknown. Our previous results showed that FMR1 expression was up-regulated in CRC, and its expression level was significantly positively correlated with tumor size, degree of differentiation, TNM stage and metastasis in CRC patients. It is a potential molecular marker of CRC. Meanwhile, using gain- and loss-of-function experiments, our data clearly indicated that FMR1 could promote CRC cell proliferation and metastasis in vitro and in vivo. Thus, our data confirmed that FMR1 exerts oncogenic activity to promote cell proliferation and metastasis in CRC.

To explore the underlying mechanism of FMR1 in the proliferation and metastasis of CRC, we used GSEA to analyze the enrichment of related genes with higher FMR1 expression. The results showed an enrichment of the ERBB-signaling pathway and its downstream pathways including the ERBB1 downstream pathway, Wnt_CANONICAL pathway, and PI3KCI_AKT pathway. EGFR is a key molecule of the ERBB-signaling pathway. The protein encoded by EGFR gene is a transmembrane glycoprotein, which is a member of the protein kinase superfamily. This protein is a receptor for members of the epidermal growth factor family. The EGFR protein is a cell surface protein involved in cell proliferation. In the development of tumors, abnormal activation of EGFR may initiate the related signaling pathways such as an ERBB-signaling pathway and the downstream ERBB pathways including RAS/ERK, PI3K/AKT or Wnt signaling (myc), ultimately promoting tumor occurrence and progression [30–35]. In the present study, we demonstrated that FMR1 promoted the activation of these signaling pathways. Meanwhile, rescue experiments showed that EGFR is required for the FMR1-mediated cell proliferation and metastasis of CRC in vitro and in vivo.

At present, more than a hundred chemical modifications have been discovered for human RNA, among which the m^6^A is quite universal in both mRNA and non-coding RNA [36, 37]. It has been reported that aberrant regulation of m^6^A modification found in CRC tissues is crucial for tumorigenesis and progression. The m^6^A regulators and m^6^A-related RNAs may become promising biomarkers and prognosis predictors as well as therapeutic targets [38]. In recent years, studies have demonstrated that FMR1 protein can recognize the m^6^A modification site, and the site contains two basic sequences, “GGAC” and “ACU” [39]. By recognizing m^6^A modification sites, FMR1 can stabilize its targeting mRNA and promote its nuclear export [40, 41]. Investigations have confirmed that there are m^6^A modification sites in EGFR mRNA, and the expression of EGFR could be regulated by m^6^A modification [42–44]. We predicted the m^6^A modification sites that FMR1 could recognize in the full-length mRNA sequence of EGFR and verify it by using dual-luciferase assay, RNA EMSA, RIP-qPCR, and meRIP-qPCR. The results showed that FMR1 can recognize the m^6^A-modification site in EGFR mRNA and bind to it. The meRIP-qPCR also indicates that m^6^A-modification site in EGFR mRNA is METTL3 dependent, for the m^6^A level in EGFR mRNA increased when m^6^A writer METTL3 is overexpressed. Moreover, we used functional experiments to verify that the effects of FMR1 on CRC progression depended on the m^6^A-modification. Rescue experiments showed that EGFR and METTL3 are required for the FMR1-mediated cell proliferation and metastasis of CRC, and the expression of FMR1 in CRC tissues was positively correlated with that of EGFR and METTL3. FMR1 and METTL3 just work jointly to regulate the expression of EGFR and its downstream pathway, however, they could not regulate each other (Supplementary Fig. S4d).

As an oncogene, FMR1 will become the target of anticancer drugs in the future. At present, the main therapeutic drugs for oncogenes include monoclonal antibodies (membrane proteins), small molecule inhibitors, and gene therapy. In recent years, gene therapy has become a research hotspot due to its strong targeting and low cost [45]. The main problem facing gene therapy is finding a suitable drug carrier [46]. Our research team focused on the development and application of RNA-interference drugs in the early stage and selected attenuated *Salmonella* as a carrier for preliminary treatment attempts. After clarifying the role of FMR1 as an oncogene in CRC, we conducted preliminary attempts using RNA interference drugs carried by attenuated *Salmonella*. The results showed that the use of RNA interference technology to inhibit FMR1 expression can effectively inhibit CRC in vivo, showing good application prospects.

Our research demonstrated that FMR1 expression levels are significantly higher in human CRC patients than that in matched normal tissues, and it is significantly correlated with the advanced progression of CRC patients. In addition, we revealed that FMR1 could recognize m^6^A modification site on EGFR mRNA, stabilize its mRNA, increase protein expression, activate its downstream signaling pathways, and mediate CRC progression. This process is completed together with an m^6^A modification writer METTL3 (Fig. 7h). Thus, FMR1 might be a target for the prediction and therapy of CRC.

## Supplementary information

Supplementary data

AJ Checklist

Original Data File

## Data Availability

The datasets used during the current study are available from the corresponding author on reasonable request.
