# Supplementary material for: FMR1 promotes the progression of colorectal cancer cell by stabilizing EGFR mRNA in an m6A-dependent manner
Source: Cell Death Dis. 2022 Nov 8;13(11):941. doi: 10.1038/s41419-022-05391-7 (PMC9643526; doi:10.1038/s41419-022-05391-7)
Supplement: Supplementary file 1 — Supplementary data [file 41419_2022_5391_MOESM1_ESM.docx]

**Supplementary material**

**Supplementary materials and methods**

**Real-time Quantitative PCR (RT-qPCR)**

Total RNA was extracted from tissues and cells using TRIzol reagent (Thermo Fisher Scientific, Inc.) according to the manufacturer’s protocol. RNA was reverse transcribed to cDNA using a PrimeScriptTM RT reagent Kit with gDNA Eraser (Takara Bio, Inc., Otsu, Japan). RT-qPCR analysis was performed using SYBR-Green I (Takara Bio, Inc.) and each experiment was performed in triplicate. The RT-qPCR reaction mixture contained 10 µl SYBRGreen RT-qPCR mastermix, 0.5 µl forward primer (10 µM), 0.5 µl reverse primer (10 µM), 1 µl cDNA and 8 µl RNase/DNase free ddH2O. The mixture was centrifuged (4°C, 1,000 × g, 10 sec) and then placed in an ABI 7500 fluorescence quantitative PCR instrument (Applied Biosystems; Thermo Fisher Scientific, Inc.). The procedures for the RT-qPCR reaction were as follows: Pre-denaturation at 95°C for 1 min; denaturation at 95°C for 15 sec; annealing and extension at 60°C for 34 sec; 40 cycles. The results were normalized to the expression of GAPDH using the 2^-ΔΔCt^ method as previously described [1]. The sequences of the primers were as listed in Supplementary Table S1.

**Western blot (WB)**

Total proteins were extracted from CRC cells. The cells were lysed in cold lysis buffer (60 mM Tris-HCl at pH 7.4, 150 mM NaCl, 0.25% SDS and 1% Tergitol-type NP-40) containing 10 mM NaF, 1 mM Na_3_VO_4_ and complete protease inhibitor (Roche Diagnostics, Basel, Switzerland) for 30 min on ice, and were then centrifuged at 10,000 × g at 4°C for 15 min as previously described. A bicinchoninic acid protein assay was used to determine protein concentration. The proteins were subjected to SDS-PAGE and transferred onto a PVDF membrane. The PVDF membrane was subsequently blocked in PBST solution containing 5% non-fat milk and incubated at 4°C overnight with specific antibodies. The next day, membranes were incubated with the corresponding HRP-conjugated secondary antibody (dilution 1:5,000; ABclonal Biotech Co., Ltd., Woburn, MA, USA). Subsequently, the membranes were detected using Pierce ECL Western Blotting Substrate (Thermo Scientific, USA)(14).

**Immunohistochemistry (IHC)**

The tissue specimens were fixed in 10% formalin, embedded in paraffin, and sectioned consecutively at 3 μm thickness using a rotary microtome for IHC. The tissue sections were deparaffinized, rehydrated, heated in 0.01M sodium citrate buffer, pH6.0 for antigen retrieval, and incubated in 3% H_2_O_2_ to inhibit endogenous peroxidase activity, followed by incubated with primary antibody at 4°C overnight. On the next day, the sections were washed and incubated with the secondary antibody for 30 minutes at room temperature. Finally, the slides were developed using a DAB chromogen kit and counterstained with Mayer’s hematoxylin. The primary antibody anti-EMB was purchased from Cusabio Technology LLC, Shanghai, China and was diluted 100 times in this study. The anti-Ki67 was purchased from Bioworld Technology Inc., St. Louis Park, MN, USA and was used with 1:100 dilution. The total immunostaining score was calculated as the sum of the percentage positivity of stained tumor cells and the staining intensity. The percentage positivity was scored 0-3: 0, <10%; 1, 10-30%; 2, 31-50%; and 3, >50%. The staining intensity was scored 0-3; 0, no staining; 1, weakly stained; 2, moderately stained; and 3, strongly stained. The percentage positivity of cells and staining intensity were decided in a double-blinded manner. Then, the score of EMB or Ki67 expression was calculated as the percentage positivity score × staining intensity score, which ranged between 0 and 9. The final expression level of EMB was defined as ‘low’ (0-4) and ‘high’ (5-9) [2].

**Cell counting kit-8 (CCK-8) assay**

The indicated cells were seeded on 96-well plates (1×10^3^ cells/well). Then the cell proliferation assay was performed over 5 days every 24 h. 10 μl CCK-8 reagent (Dojindo Laboratories, Japan) was added to each well and then the plate was incubated for 2 h at 37°C. After that, the absorbance was measured at 450 nm using a Vmax microplate spectrophotometer (Molecular Devices, Sunnyvale, CA). Each sample was assayed in triplicate and repeated 3 times independently [3].

**Colony Formation Assay**

The indicated cells were trypsinized and seeded on 6-well plates (200 cells/well) and cultured for 2 weeks. The colonies were stained with Hematoxylin for 30 min after fixation with 4% paraformaldehyde for 30 min. The number of colonies, defined as > 50 cells/colony, was counted. Three independent experiments were performed [3].

**Transwell assay**

1×10^5^ cells in culture medium containing 1% fetal bovine serum were plated on the top side of Boyden chamber (BD, Bedford, MA), and culture medium with 10% fetal bovine serum was added in the lower chamber as a chemoattractant. Then the cells incubated at 37°C for 24-48 hours. Invaded and migratory cells on the lower membrane surface were fixed in 4% paraformaldehyde for 30 minutes. After removal of cells inside the upper chamber with cotton swabs, the invaded and migratory cells were stained with Giemsa for counting (3 random 100× fields per well). Cell counts were expressed as the mean number of cells per field of view. Three independent experiments were performed, and the data are presented as mean ± SD.

**Wound healing assays**

5 × 10^5^ cells/well were seeded into 6-well plates. When the cell density reached 80 to 90%, a scratch was made in the monolayer in the middle of the well with a 10 μl pipette tip. The tip was kept perpendicular to the bottom of the well to obtain a straight gap, and the detached cells were washed away. Wound healing within the same scraped line was then observed and photographed at the indicated time points (0 h, 24 h, 48 h, and 96 h). Each experiment was repeated three times.

**References**

1. Li X, Wang J, Zhang C, Lin C, Zhang J, Zhang W, et al. Circular RNA circITGA7 inhibits colorectal cancer growth and metastasis by modulating the Ras pathway and upregulating transcription of its host gene ITGA7. J Pathol. 2018;246:166-79.

2. Gu Y, Wang Q, Guo K, Qin W, Liao W, Wang S, et al. TUSC3 promotes colorectal cancer progression and epithelial-mesenchymal transition (EMT) through WNT/β-catenin and MAPK signalling. J Pathol. 2016;239:60-71.

3. Hu YH, Ma S, Zhang XN, Zhang ZY, Zhu HF, Ji YH, et al. Hypermethylation Of ADHFE1 Promotes The Proliferation Of Colorectal Cancer Cell Via Modulating Cell Cycle Progression. Onco Targets Ther. 2019;12:8105-15.

**Supplementary Table S1. Primer sequences used for RT-qPCR (5’ to 3’)**

| Gene | Sense primer（5’→3’） | Anti-sense primer（5’→3’） |
| --- | --- | --- |
| FMR1 | GTCACCTCAAAGCGAGCA | ATTAGCACCATGAGTACCAA |
| EGFR | AAAGTTAAAATTCCCGTCGCTA | CCATCACGTAGGCTTCATCGAG |
| METTL3 | CTACGGAATCCAGAGGCAGCATTG | GCGTGGAGATGGCAAGACAGATG |
| GAPDH | GGUGACUAUUCAACCGCAUTT | AUGCGGUUGAAUAGUCACCTT |

**Supplementary Table S2. Antibodies used for Western blot or IHC.**

| Gene | Manufacturer | Dilution |
| --- | --- | --- |
| FMR1 | Proteintech, USA | 1:1000 for WB; 1:100 for IHC |
| bax | Proteintech, USA | 1:500 for WB |
| p21 | Proteintech, USA | 1:500 for WB |
| p27 | Proteintech, USA | 1:500 for WB |
| Cyclin D1 | Proteintech, USA | 1:1000 for WB |
| GAPDH | Proteintech, USA | 1:50000 for WB |
| EGFR | Proteintech, USA | 1:5000 for WB; 1:100 for IHC |
| RAS | Proteintech, USA | 1:10000 for WB |
| MEK | Proteintech, USA | 1:5000 for WB |
| p-MEK | Proteintech, USA | 1:1000 for WB |
| ERK | Proteintech, USA | 1:1000 for WB |
| p-ERK | Proteintech, USA | 1:500 for WB |
| AKT | Proteintech, USA | 1:500 for WB |
| p-AKT | Proteintech, USA | 1:500 for WB |
| myc | Proteintech, USA | 1:5000 for WB |
| METTL3 | Proteintech, USA | 1:1000 for WB; 1:100 for IHC |
| Ki67 | Zhongshan Golden Bridge Biotechnology, China | 1:100 for IHC |

**Supplementary Table S3. Sequences used for shRNA FMR1 (5’ to 3’)**

|  | 5’→3’ |  |
| --- | --- | --- |
| shFMR1-1 | GATCCCTTTCTACAAGGCATTTGTAATTCAAGAGATTACAAATGCCTTGT  AGAAAGTTTTTTGGAAA **(Sense)**  AGCTTTTCCAAAAAACTTTCTACAAGGCATTTGTAATCTCTTGAATTAC  AATGCCTTGTAGAAAGG **(Anti-sense)** | |
| shFMR1-2 | GATCCGTTGGTGGTTAGCTAAAGTGATTCAAGAGATCACTTTAGCTAAC  CACCAACTTTTTTGGAAA **(Sense)**  AGCTTTTCCAAAAAAGTTGGTGGTTAGCTAAAGTGATCT CTTGAATCA  CTTTAGCTAACCACCAACG **(Anti-sense)** | |
| shFMR1-3 | GATCCGGAGAGATTACAAATTGATGATTCAAGAGATCATCAATTTGTAA  TCTCTCC TTTTTTGGAAA **(Sense)**  AGCTTTTCCAAAAAA GGAGAGATTACAAATTGATGATCTCTTGAATCA  TCAATTTGTAATCTCTCCG **(Anti-sense)** | |

**Figure S1**

**
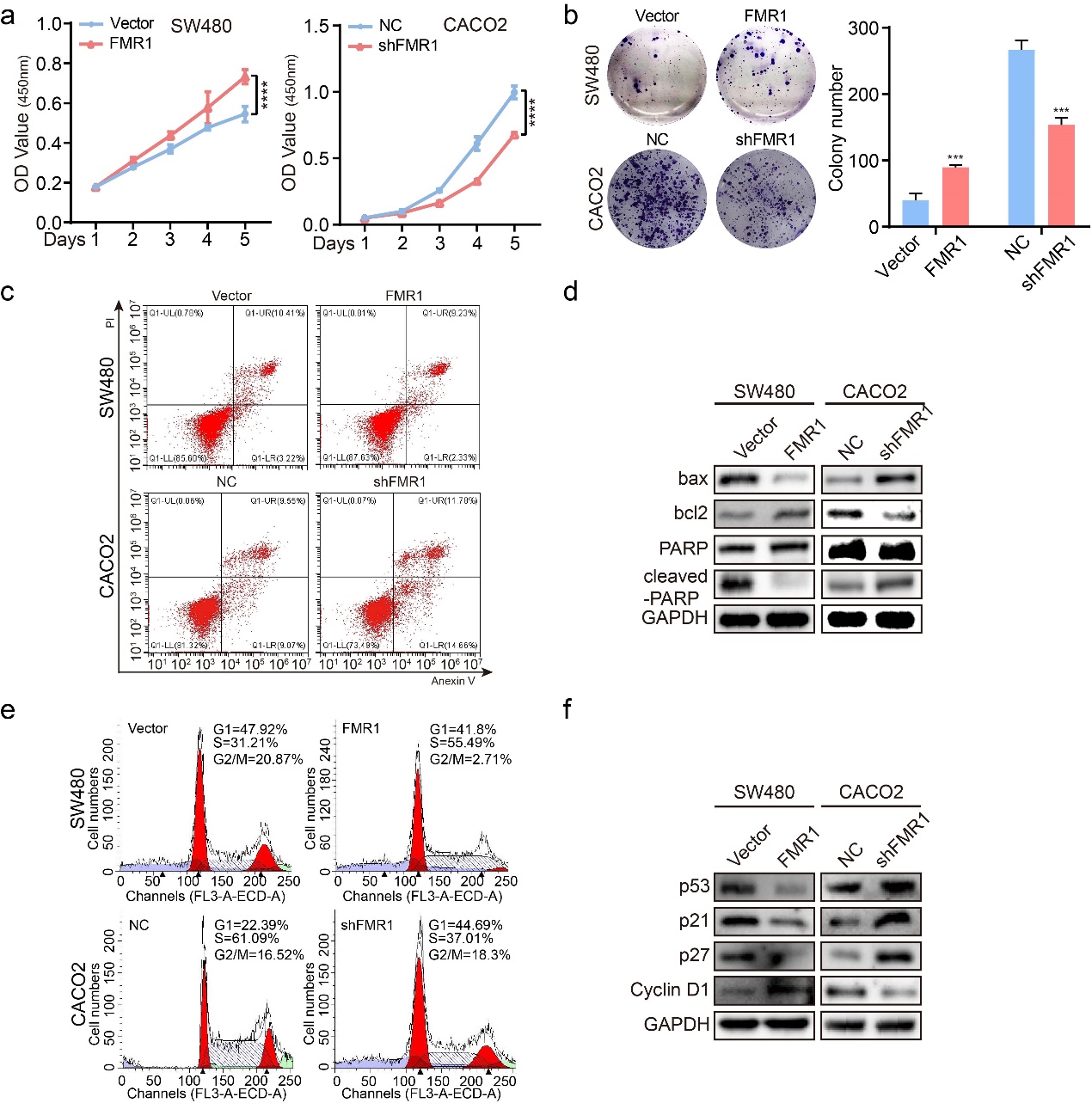
**

**Fig. S1 FMR1 regulates proliferation, apoptosis and cell cycle of in vitro.** a, CRC cell proliferation was analyzed by CCK8 assays. b, Representative results of colony formation; the numbers of colonies containing>50 cells were scored. The number of colonies counted was of an entire well and the error bars represent mean ± SD from three independent experiments. c, Apoptosis assay by flow cytometry. Annexin-positive/PI-negative (right lower quadrant) cells were analyzed for apoptosis rate. d, Western blot was used to test the molecular markers of apoptosis. e, Flow-cytometry analyses of the cell cycle of the indicated CRC cells. f, Western blot was used to test the molecular markers of cell cycle.

**Figure S2**


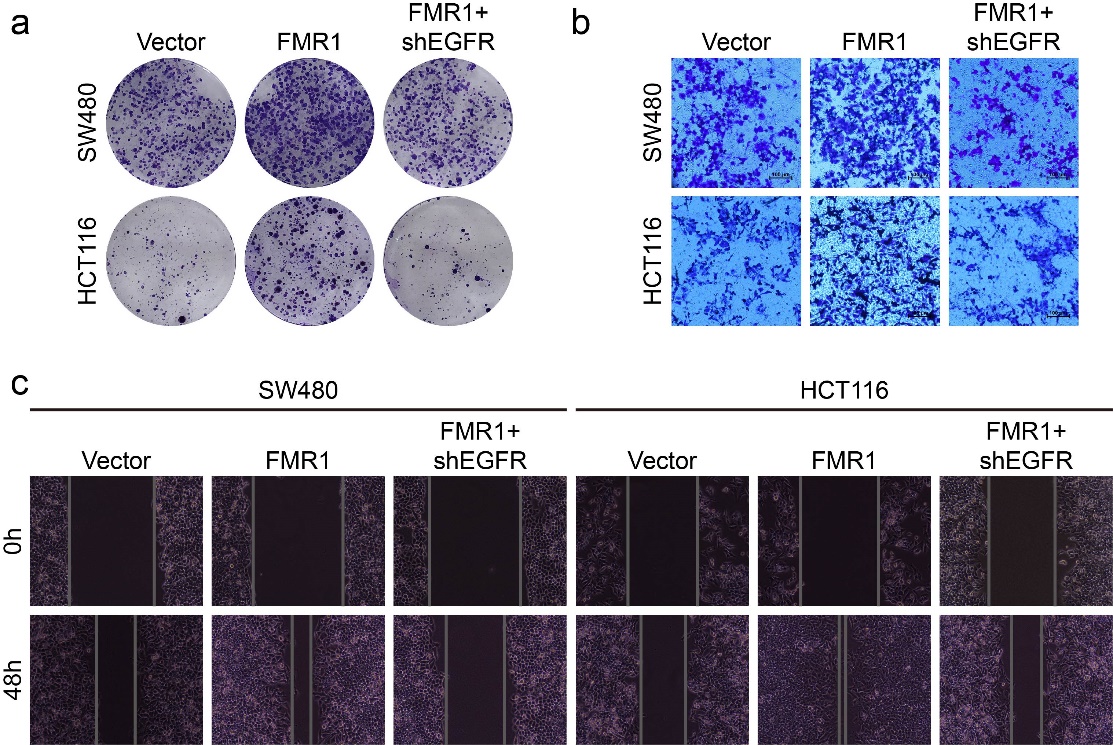


**Fig. S2 FMR1 promotes the progression of CRC by upregulating EGFR in vitro.** a, Representative results of colony formation. b Representative results of transwell assays. c, Representative results of wound healing assays.

**Figure S3**


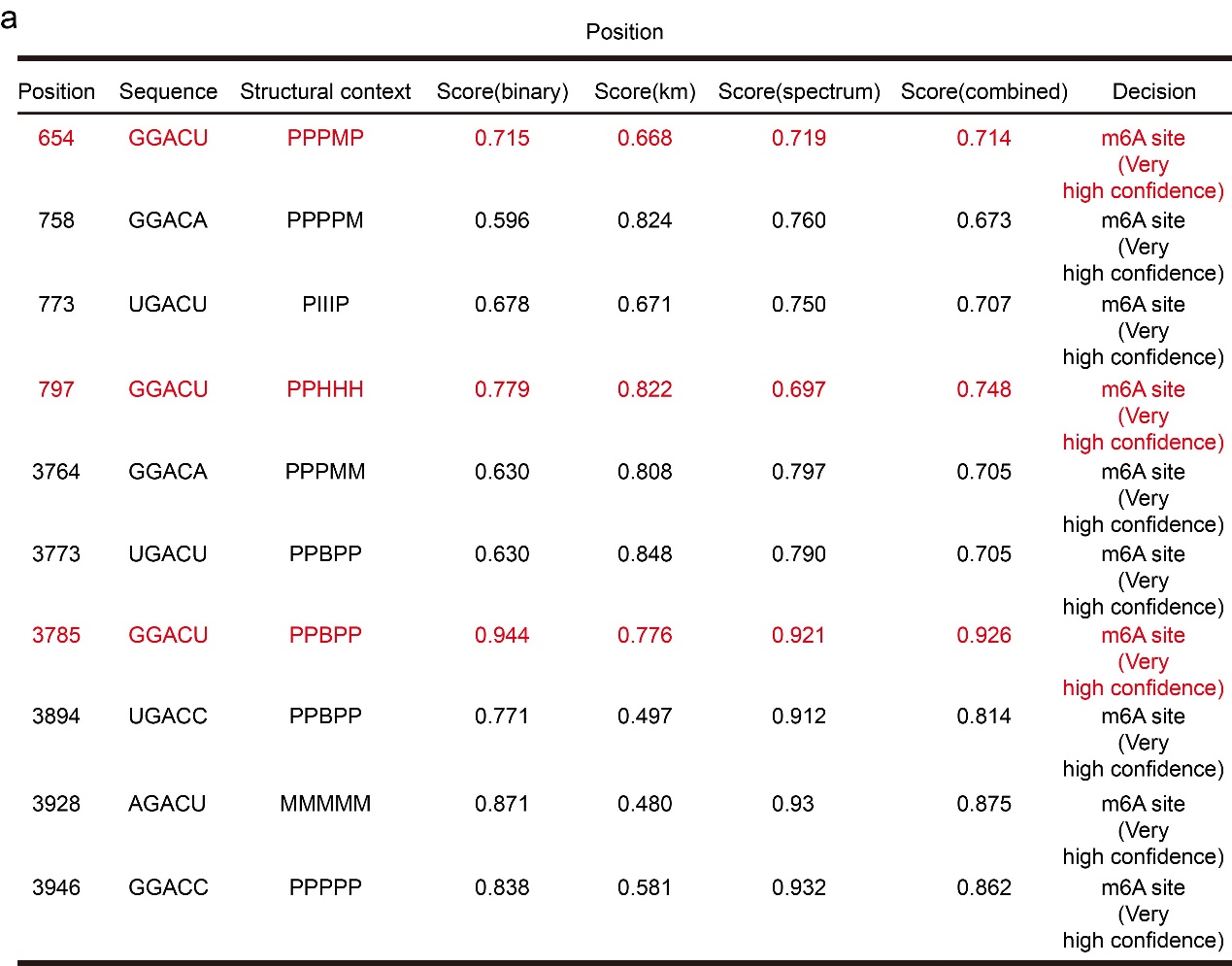


**Fig. S3 Prediction of specific binding sites recognized by FMR1 protein on the EGFR mRNA sequence.** a, The scores of specific binding sites recognized by FMR1 protein on the EGFR mRNA sequence.

**Figure S4**


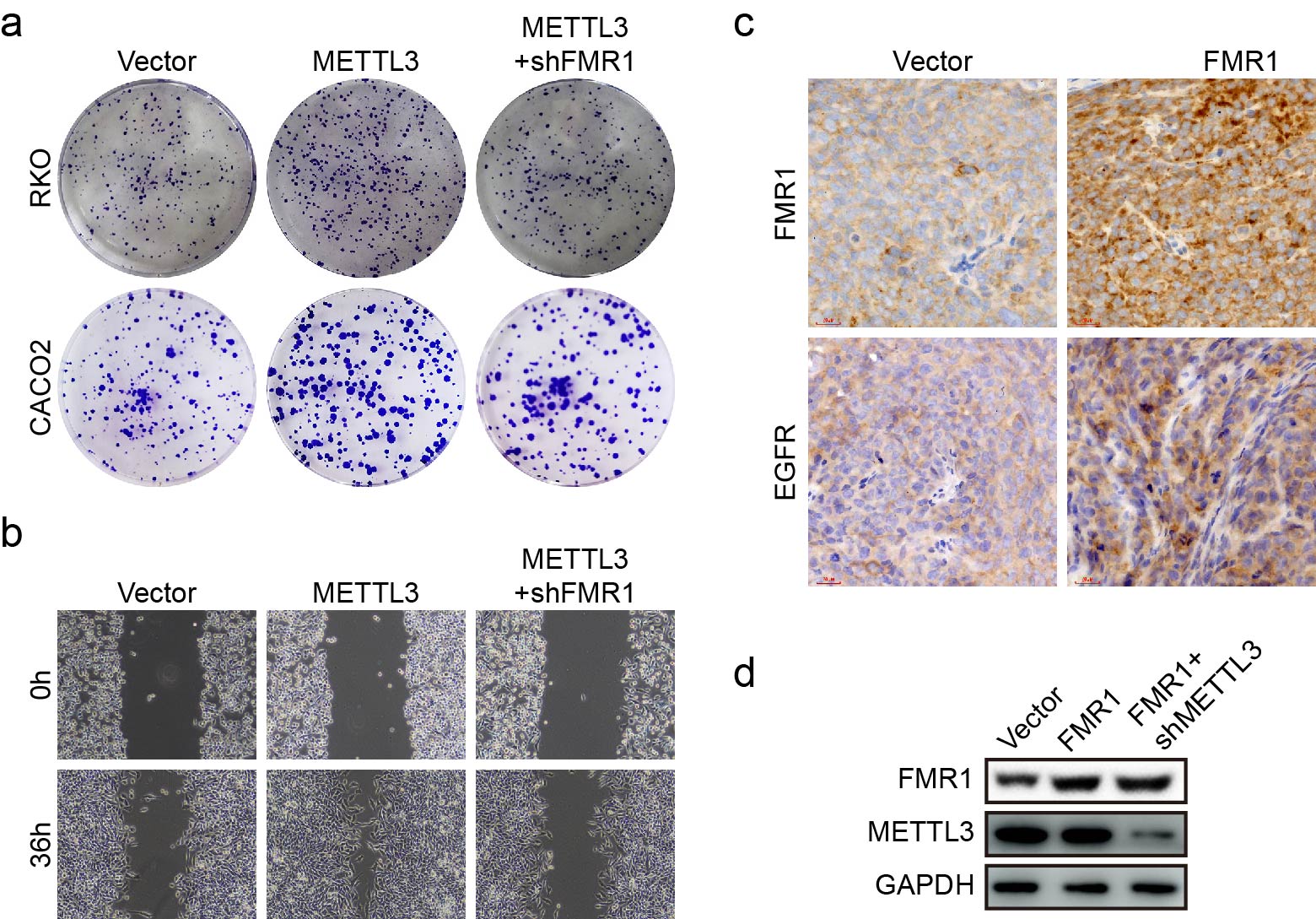


**Fig. S4 FMR1 works together with METTL3 and EGFR in the progression of CRC.** a, Representative results of colony formation. b, Representative results of wound healing assays. c, The images of IHC for FMR1 and EGFR in xenografts were shown. d, Western blot was used to test the expression of FMR1 and METLL3 when FMR1 was overexpressed or METTL3 was knocked.
